# Proanthocyanidins-Mediated Nrf2 Activation Ameliorates Glucocorticoid-Induced Oxidative Stress and Mitochondrial Dysfunction in Osteoblasts

**DOI:** 10.1155/2020/9102012

**Published:** 2020-09-25

**Authors:** Liang Chen, Sun-Li Hu, Jun Xie, De-Yi Yan, She-Ji Weng, Jia-Hao Tang, Bing-Zhang Wang, Zhong-Jie Xie, Zong-Yi Wu, Lei Yang

**Affiliations:** ^1^Department of Orthopaedic Surgery, The Second Affiliated Hospital and Yuying Childrens Hospital of Wenzhou Medical University, Wenzhou 325000, China; ^2^Key Laboratory of Orthopaedics of Zhejiang Province, Wenzhou 325000, China

## Abstract

The widespread use of therapeutic glucocorticoids has increased the frequency of glucocorticoid-induced osteoporosis (GIOP). One of the potential pathological processes of GIOP is an increased level of oxidative stress and mitochondrial dysfunction, which eventually leads to osteoblast apoptosis. Proanthocyanidins (PAC) are plant-derived antioxidants that have therapeutic potential against GIOP. In our study, a low dose of PAC was nontoxic to healthy osteoblasts and restored osteogenic function in dexamethasone- (Dex-) treated osteoblasts by suppressing oxidative stress, mitochondrial dysfunction, and apoptosis. Mechanistically, PAC neutralized Dex-induced damage in the osteoblasts by activating the Nrf2 pathway, since silencing Nrf2 partly eliminated the protective effects of PAC. Furthermore, PAC injection restored bone mass and promoted the expression of Nrf2 in the distal femur of Dex-treated osteoporotic rats. In summary, PAC protect osteoblasts against Dex-induced oxidative stress and mitochondrial dysfunction via the Nrf2 pathway activation and may be a promising drug for treating GIOP.

## 1. Introduction

Dexamethasone (Dex) is a synthetic glucocorticoid (GC) used widely for treating inflammatory and autoimmune diseases [1, 2]. However, chronic Dex treatment is associated with adverse effects, including decreased bone mineral density and microarchitecture porosity, eventually leading to glucocorticoid-induced osteoporosis (GIOP) and osteonecrosis [3–5]. Currently, therapeutics for GIOP are predominantly aimed to prevent excessive bone resorption; however, this therapy does not help to restore bone mass and bone microstructure. In addition, long-term use of antiresorptive drugs, such as bisphosphonates, may lead to a decrease in bone turnover. The development of GIOP is based on the disbalance between osteogenesis and osteoclastogenesis [6]. Therefore, preventing the deleterious effects on osteoblasts has become a major issue, and the underlying mechanisms of Dex on osteoblasts remains to be unraveled. This is because endogenous GC activities are required for the maintenance of bone homeostasis [6]; however, excess GCs impede bone formation by impairing osteoblast differentiation, mineralization function, and survival [7]. The harmful effects of GC on osteoblasts are now considered as crucial factors in the development of GIOP [8, 9]. High-dose and long-term use of Dex leads to osteoblast apoptosis, which has been reported to be associated with the accumulation of reactive oxygen species (ROS) and the loss of mitochondrial membrane potential (MMP) [10, 11]. Furthermore, Dex-induced ROS accumulation and mitochondrial damage in osteoblasts exacerbate GIOP progression and bone mass loss through lipid peroxidation, inhibition of antioxidant enzymes, and increased osteoblast apoptosis [10, 11]. Moreover, an excess of oxidative stress induced by Dex has been reported to negatively impact the osteoblast differentiation and mineralization function. Thus, key to the treatment of GIOP involves reducing intracellular oxidative stress in osteoblasts.

Nuclear factor erythroid 2-related factor 2 (Nrf2) is a vital transcription factor that is dysregulated in various oxidative stress-related pathologies [12]. Since ROS accumulation accelerates the development of GIOP [13, 14] and the activation of Nrf2 can effectively reduce oxidative stress and apoptosis [15], it is a promising therapeutic target for GIOP. In addition, Ibáñez et al. observed significant bone loss in ovariectomized Nrf2^−/−^ compared to that in wild type controls [16], which points to an osteoprotective function of Nrf2 as well [17]. It combats intracellular oxidative stress by activating multiple downstream genes, such as heme oxygenase-1 (HO-1), superoxide dismutase (SOD), glutathione (GSH), and catalase (CAT) [18, 19], by binding to the upstream antioxidant response element (ARE) sequence [20].

Several plant-derived antioxidants can activate the Nrf2 pathway and protect cells against oxidative damage [21]. Proanthocyanidins (PAC) are a group of polyphenolic flavonoids present in many vegetables, flowers, fruits, and nuts [22] and exhibit antibacterial, antiviral, antioxidant, and other pharmaceutical effects [23]. In addition, several studies have shown that PAC can activate the Nrf2 pathway [24, 25]. However, their potential therapeutic effect in GIOP is still elusive. We demonstrated for the first time that PAC can suppress osteoblast apoptosis and dysfunction during the GIOP development by activating the Nrf2 pathway, indicating that PAC are a suitable therapeutic option for GIOP.

## 2. Materials and Methods

### 2.1. Reagents, Antibodies, and Media

PAC (≥96%) and Dex were purchased from Sigma-Aldrich (St. Louis, MO, USA). Primary antibodies against COL1A1, RUNX2, and OCN were from Abcam (Cambridge, UK), and those targeting Nrf2, HO-1, Bax, Bcl-2, cleaved caspase-3, *β*-actin, and lamin B were obtained from Cell Signaling Technology (Danver, MA, USA). Fetal bovine serum (FBS), Dulbecco's Modified Eagle Medium (DMEM), and penicillin/streptomycin were purchased from Gibco BRL (Thermo Fisher Scientific, Waltham, MA, USA). All other chemicals used were of analytical grade complying with tissue and cell culture standards.

### 2.2. Cell Culture

Primary osteoblasts were isolated from the calvarial bone of 1-day-old Sprague Dawley rats by continuous digestion with trypsin-collagenase [26] and cultured in complete DMEM supplemented with 1% (v/v) penicillin-streptomycin and 10% (v/v) FBS under 5% CO_2_ at 37°C. Medium was changed every other day, and the cells were passaged when 80-90% confluent. In brief, cells received the following treatment: (1) Dex group: cells were treated with 5 *μ*M Dex for 48 hours, then cultured in complete DMEM medium for another 48 hours; (2) Dex+PAC group: cells were treated with 5 *μ*M Dex for 48 hours and then cocultured with 0.1 and 1 *μ*M PAC for another 48 hours; and (3) control group: untreated osteoblasts were cultured for the same period of time as controls.

### 2.3. Cytotoxicity Assay

The effect of PAC and Dex on osteoblast viability was determined using the Cell Counting Kit-8 (CCK-8) assay (MedChemExpress LLC; Monmouth Junction, NJ, USA). The cells were seeded in a 96-well plate at the density of 5 × 10^3^ cells per well and treated with varying doses of PAC (0.01, 0.1, 1, and 10 *μ*M) with or without Dex pretreatment for 48 hours. After 48 hours, 10 *μ*l of CCK8 reagent was added to each well, and the cells were incubated for another hour. The absorbance or optical density (OD) at 450 nm was measured using the Multiskan GO microdisk spectrophotometer (Thermo Fisher Science, Waltham, MA, USA).

### 2.4. Osteogenic Differentiation of Primary Calvarial Osteoblasts

The cells were seeded in a 24-well plate at the density of 5 × 10^4^ cells per well. After having received the indicated treatment, osteoblasts were cultured in DMEM supplemented with 20 *μ*M ascorbic acid and 10 mM *β*-glycerophosphate. The media was replaced every other day. The alkaline phosphatase (ALP) activity was measured after 7 days of differentiation using the ALP Staining Kit (Beyotime Institute of Biotechnology; Jiangsu, China). For mineralization and bone nodule formation, the cells were cultured under osteogenic conditions for 21 days, fixed, and stained with Alizarin Red S (ARS) (Solarbio Science & Technology; Beijing, China).

### 2.5. Western Blotting

Total protein was extracted from cultured cells using RIPA lysis buffer (Beyotime Institute of Biotechnology) containing protease and phosphatase inhibitors (Sigma-Aldrich) for 30 min at 4°C. The nuclear and cytoplasmic protein fractions were separated using a commercial kit according to the manufacturer's instructions (Beyotime Institute of Biotechnology). The cell lysates were centrifuged, and the protein concentration in the supernatant was determined by the BCA Protein Assay Kit (Beyotime Institute of Biotechnology) as per the manufacturer's protocol. An equal amount of protein (20 *μ*g) per sample was denatured in SDS loading buffer by boiling for 5 mins and resolved on a 10-15% SDS–polyacrylamide gel. The protein bands were then transferred overnight to polyvinylidene difluoride (PVDF) membranes (Merck Millipore; Burlington, MA, USA) at 4°C. After blocking with 5% skim milk diluted in Tris-buffered saline with 0.1% Tween 20 (TBST) for 2 hours at room temperature, the blots were probed for 12 hours with the suitable primary antibodies at 4°C. The membranes were washed thrice with TBST and incubated with the corresponding HRP-conjugated secondary antibodies for 4 hours at room temperature. The positive bands were visualized by enhanced chemiluminescence on a ChemiDoc XRS+ imager (Bio-Rad; Hercules, CA, USA) and quantified by densitometry analysis using Image Lab V3.0 software (Bio-Rad). The experiment was repeated thrice.

### 2.6. Immunofluorescence

Primary calvarial osteoblasts were cultured on glass coverslips under suitable conditions, fixed with 4% paraformaldehyde (PFA) for 15 min at room temperature, and then permeabilized with 0.5% (v/v) Triton X-100 in PBS for 20 mins. Nonspecific antibody binding was blocked with 1% (w/v) goat serum albumin for 1 hour at room temperature, followed by overnight incubation with primary antibodies in 0.2% BSA-PBS at 4°C with gentle shaking. The cells were washed with PBS and incubated with fluorescence-conjugated secondary antibodies for 1 hour at room temperature in the dark. After counterstaining with DAPI for 5 mins at room temperature, the cells were viewed under the Olympus BX53 fluorescence microscope (Olympus Life Science; Tokyo, Japan). Integrated optical density (IOD) of the positively stained samples was calculated using Image-Pro Plus software (Media Cybernetics, Inc; Rockville, MD, USA).

### 2.7. Transfection

The siRNA targeting Nrf2 was obtained from the RiboBio company. The osteoblasts were transfected with the Nrf2 or control siRNA according to the manufacturer's protocol.

### 2.8. Caspase-3 Activity Assay

The caspase-3 activity in the suitably treated cells was measured using a Caspase-3 Activity Assay Kit (Nanjing Jiancheng Bioengineering Institute, Nanjing, China) as per the manufacturer's instructions. In general, treated cells were washed 3 times with PBS and resuspended in lytic buffer and placed on ice for 20 minutes. The lysis solution was then added to the reaction buffer containing Ac-DEVD-pNA and incubated at 37°C for 2 hours. Subsequently, the absorbance of yellow pNA from its corresponding precursor was measured by a spectrometer at 405 nm. The caspase-3 activity was calculated as the ratio of the value obtained from the treated cells to the value obtained from untreated control cells.

### 2.9. TUNEL Assay

Apoptosis was measured using an in situ Cell Death Detection Kit (Roche, South San Francisco, CA, USA) according to the instruction of the manufacturer. The number of the apoptotic cells was counted in three random fields under a fluorescence microscope (Olympus Life Science; Tokyo, Japan).

### 2.10. Quantification of SOD, CAT, and GPx Activities

The suitably treated cells were washed twice with PBS and lysed on ice for 30 minutes. The activities of SOD, CAT, and GPx in the lysates were detected using commercial assay kits (Jiancheng Biotechnology, Nanjing, China) as per the manufacturer's instructions.

### 2.11. Intracellular ATP and ROS Assay

Intracellular ATP and ROS levels were measured using the ATP Assay and Reactive Oxygen Species Assay Kits according to the manufacturer's instructions (Beyotime, Shanghai, China).

### 2.12. Mitochondrial Function Assays

The mitochondrial membrane potential (MMP) and superoxide ion levels in the suitably treated osteoblasts were determined by, respectively, staining with JC-1 and MitoSox as per the manufacturer's guidelines (Beyotime, Shanghai, China). Stained cells were observed under a fluorescence microscope (Olympus Life Science; Tokyo, Japan).

### 2.13. Transmission Electron Microscopy (TEM)

Treated primary calvarial osteoblasts were fixed in 2.5% glutaraldehyde for 12 hours at 4°C, postfixed in 2% osmium tetroxide for 1 hour, and stained with 2% uranyl acetate for 1 hour at room temperature. Then, cells were dehydrated in cold graded ethanol series (30%, 50%, 70%, 80%, 90%, 100% ethanol; 10 mins each) and washed three times with 100% acetone (20 min each time with gentle rocking). Next, cells were embedded in araldite epoxy resin, and semithin sections were cut and stained with toluidine blue. TEM images were captured on a Hitachi Field Emission Transmission Electron Microscope (Hitachi High-Technologies Corp.; Tokyo, Japan).

### 2.14. Establishment of a Rat Model of GIOP

All animal experiments were approved by the Animal Ethics Committee of The Second Affiliated Hospital and Yuying Children's Hospital of Wenzhou Medical University (Wenzhou, China) and conducted pursuant to the criteria outlined in the Guide for the Care and Use of Laboratory Animals (NIH, Bethesda, MD, USA). Forty-five 3-month-old SD male rats were purchased from Shanghai Laboratory Animal Center (SLACCAS; Shanghai, China) and housed under SPF conditions at 22-25°C and 12 hours of the light/dark cycle. All animals had free access to tap water and a standard rodent diet consisting of 2.5% casein, 0.8% phosphorus, 1% calcium, 70-80% carbohydrates, and 5% fat (Provimi Kliba AG, Kaiseraugst, Switzerland). After one week of acclimatization, the rats were randomly divided into the vehicle control (sham group), untreated GIOP, and PAC-treated GIOP groups (*n* = 15 each). GIOP was induced by daily intraperitoneal injections of 5 mg/kg Dex for 4 weeks as previously described [4], and the rats in the sham group received daily injections of PBS during the same period. After 4 weeks of Dex treatment, the animals showing signs of osteoporosis as per X-ray radiograph imaging were treated with the vehicle or PAC (10 mg/kg) for another 8 weeks. The rats were sacrificed after the treatment regimen, and the bilateral femurs were removed and fixed in 4% paraformaldehyde. All procedures were conducted in strict accordance with the Animal Care and Use Committee of Wenzhou Medical University (Wenzhou, China).

### 2.15. Micro-CT Analysis

Microstructural analysis of the distal femoral bones was performed using a cabinet cone-beam micro-CT system and associated software (*μ*CT 50, Scanco Medical; Brüttisellen, Switzerland). The images were acquired at 70 kV, 200 *μ*A, and a spatial resolution of 14.8 mm in all directions. Three-dimensional reconstructed images were generated, and the volume of interest (VOI) included the trabecular compartment 2 mm below the highest point of the growth plate to the distal 100 CT slices. Quantitative bone parameters assessed within the VOI included the percentage bone volume to tissue volume (BV/TV), the mean trabecular thickness (Tb.Th, mm), the mean trabecular number (Tb.N, 1/mm), the mean trabecular separation (Tb.Sp, mm), and the mean connective density (Conn.D, 1/mm^3^).

### 2.16. Histology, Immunohistochemistry (IHC), and Immunofluorescence

The fixed femurs were decalcified in 10% EDTA for 4 weeks, dehydrated through an ethanol gradient (70 to 100%), cleared with xylene, and embedded in paraffin. Hematoxylin-eosin and Mason trichrome staining were performed on longitudinal 4 *μ*m-thick serial slices according to the manufacturer's instructions. For IHC, 6 *μ*m-thick sections were incubated with the anti-Nrf2 primary antibody, followed by the horseradish peroxidase detection system according to the manufacturer's protocol (Vector Laboratories; Burlingame, CA, USA). For immunofluorescence staining, the sections were incubated overnight with the anti-Nrf2 antibody at 4°C, followed by the fluorochrome-conjugated secondary antibody at 37°C in the dark for 1 hour. After counterstaining with DAPI for 1 minute, the tissue sections were observed under a Olympus BX53 light/fluorescence microscope equipped with a Olympus DP71 digital camera (Olympus Life Science). The in situ Nrf2 expression was analyzed using Image-Pro Plus software.

### 2.17. Statistical Analyses

All statistical analyses were performed using the GraphPad Prism software (San Diego, CA, USA), and the data was presented as the mean ± standard error of mean (SEM) from at least three experimental repeats. Two-tailed Student's *t*-test was used to compare means between two groups, and one-way ANOVA with Bonferroni or Dunnett corrections was used for multiple comparisons. *p* < 0.05 was considered statistically significant.

## 3. Results

### 3.1. PAC Alleviated Dex-Induced Damage in Osteoblasts

**Figure 1:**
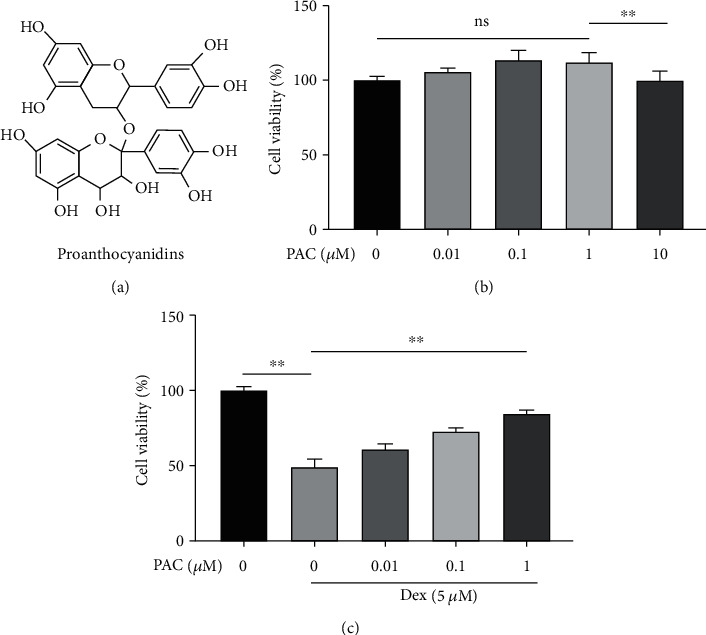
Effects of PAC on osteoblast viability. (a) The chemical structure of PAC. (b) Percentage of viable cells receiving treatment with different PAC doses for 48 h. (c) Percentage of viable cells pretreated with Dex with/without different concentrations of PAC. The data in the figures represent the averages ± SEM of 5 times in duplicates. ^∗∗^*p* < 0.01 versus the relative groups.

**Figure 2:**
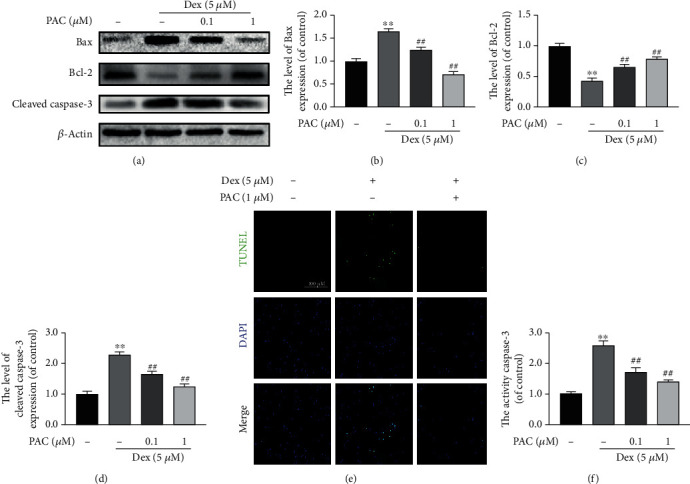
PAC suppressed Dex-induced apoptosis in osteoblasts. (a–d) Expression levels of cleaved caspase-3, Bcl-2, and Bax in the differentially treated osteoblasts. (e) Representative fluorescence images showing coculture with PAC (1 *μ*M) reduced Dex-induced osteoblast apoptosis. (f) Caspase-3 activity in the differentially treated osteoblasts. The data in the figures represent the averages ± SEM of 5 times in duplicates. ^∗∗^*p* < 0.01 versus the untreated group, ^##^*p* < 0.01 versus the Dex group.

PAC are electrophilic flavanols found predominantly in dark-colored plants (Figure 1(a)). We treated the primary osteoblasts with varying doses of PAC, and 1 *μ*M was the highest nontoxic concentration (Figure 1(b)). Furthermore, PAC rescued the impaired viability of the Dex-treated cells in a dose-dependent manner (Figure 1(c)). Based on these results, 0.1 and 1 *μ*M PAC were used for subsequent experiments. Dex treatment significantly increased the number of TUNEL positive apoptotic osteoblasts, which was alleviated by PAC (Figure 2(e)). Consistent with these findings, the proapoptotic caspase-3, cleaved caspase-3, and Bax were upregulated, the antiapoptotic Bcl-2 was downregulated in Dex-treated cells (Figures 2(a)–2(d), (f)), and these proteins returned to normal levels after PAC treatment. Taken together, our results indicated that PAC were able to ameliorate Dex-induced damage in osteoblasts.

### 3.2. PAC Neutralized Dex-Induced Oxidative Stress in Osteoblasts

**Figure 3:**
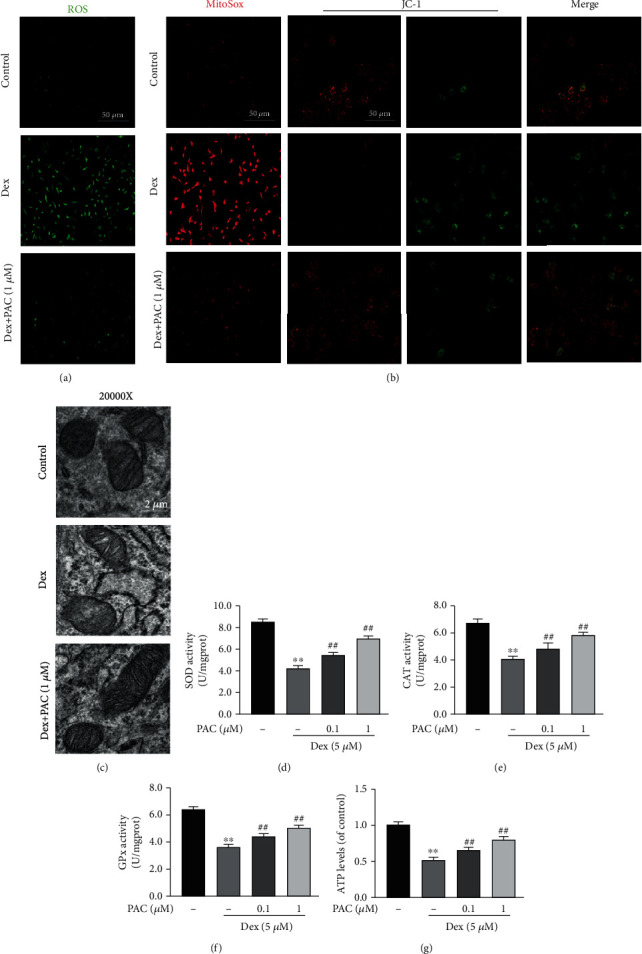
PAC neutralized Dex-induced oxidative stress and mitochondrial dysfunction in osteoblasts. (a) Representative images showing the ROS level in the differentially treated osteoblasts. (b) Representative images showing MitoSox and JC-1 intensities in the differentially treated osteoblasts. (c) Representative images showing mitochondrial morphology in the differentially treated osteoblasts. (d–f) The activities of SOD, CAT, and GPx in Dex-treated cells with/without PAC. (g) The ATP levels in the differentially treated osteoblasts. The data in the figures represent the averages ± SEM of 5 times in duplicates. ^∗∗^*p* < 0.01 versus the untreated group, ^##^*p* < 0.01 versus the Dex group.

The molecular basis of GIOP is the decreased osteogenic ability caused by high oxidative stress levels and mitochondrial dysfunction in the osteoblasts. Therefore, we also evaluated the effect of PAC on ROS production, mitochondrial dysfunction, and antioxidant enzyme activity in the Dex-treated osteoblasts. Compared to the untreated cells, Dex exposure increased ROS accumulation, which was neutralized by PAC pretreatment (Figure 3(a)). The MMP and mitochondrial ROS were, respectively, detected using JC-1 and MitoSox probes. The Dex-treated cells had significantly lower MMP and elevated superoxide anion content compared to the control cells, and both were restored to near-physiological levels by PAC treatment (Figure 3(b)). This was further corroborated via TEM analysis, showing a deteriorating morphology of mitochondria after Dex exposure, which was restored by PAC treatment (Figure 3(c)). In addition, Dex-treated osteoblasts, coculture with PAC, also restored the activities of SOD, CAT, and GPx (Figures 3(d)–3(f)), along with ATP levels (Figure 3(g)).

### 3.3. PAC Restored the Differentiation and Mineralization of Dex-Treated Osteoblasts

**Figure 4:**
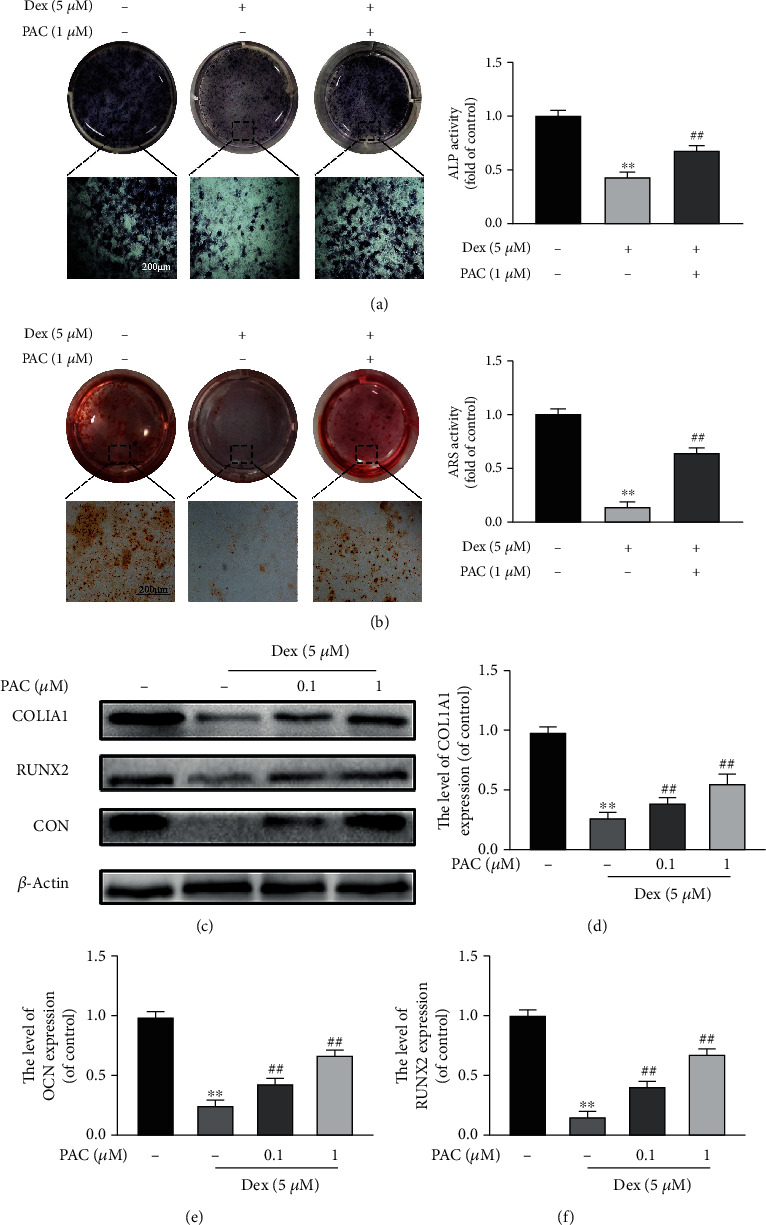
PAC restored differentiation and mineralization of Dex-treated osteoblast. Representative images showing (a) ALP staining on the 7^th^ day and (b) ARS staining on the 21^st^ day of osteogenic induction in differentially treated osteoblasts. (c–f) Expression levels of RUNX2, COL1A1, and OCN in the differentially treated osteoblasts. Data are the average ± SEM of 3 independent experiments. ^∗∗^*p* < 0.01 versus the untreated group, ^##^*p* < 0.01 versus the Dex group.

The osteogenic function of osteoblasts is inhibited by oxidative stress and mitochondrial dysfunction [4, 5]. Therefore, we next assessed the effect of PAC on the early differentiation and mineralization of the Dex-treated cells. Dex markedly reduced osteogenic differentiation, as measured by the ALP activity after 7 days of culture, and decreased the formation of calcium nodules by the 21^st^ day (Figures 4(a) and 4(b)). However, cotreatment with PAC restored the ALP activity and the extent of mineralization in the Dex-treated osteoblasts, which also correlated with an upregulation of the osteogenic transcription factors RUNX2, COL1A1, and OCN after 7 days of osteogenic induction (Figures 4(c)–4(f)). Taken together, PAC protect osteoblasts against the inhibitory effects of Dex.

### 3.4. The Osteoprotective Effects of PAC Are Mediated by the Nrf2 Pathway

**Figure 5:**
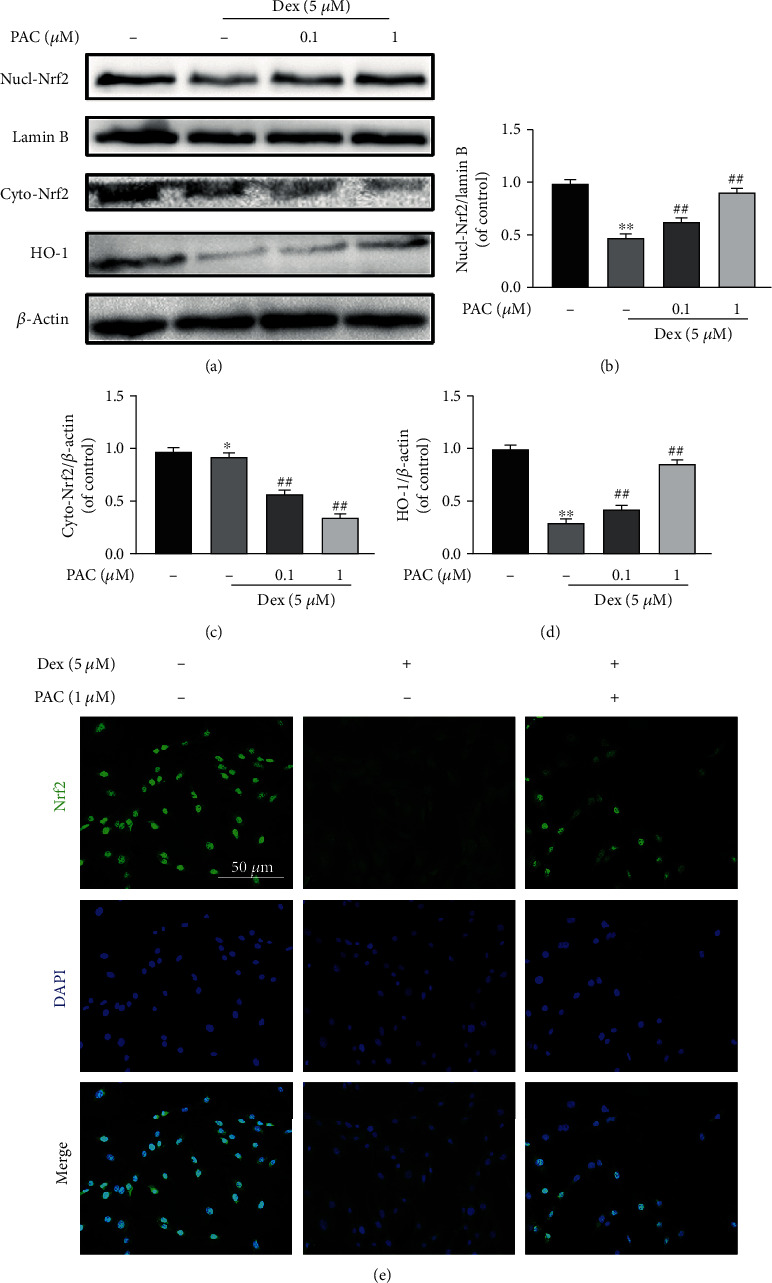
PAC activated the Nrf2 pathway in osteoblasts. (a–d) Immunoblot showing Nrf2 and HO-1 levels in the differentially treated osteoblasts. (e) Representative fluorescence images showing Nrf2 localization in the differentially treated cells. Data are the averages ± SEM of 3 independent experiments. ^∗^*p* < 0.05, ^∗∗^*p* < 0.01 versus the untreated group, ^##^*p* < 0.01 versus the Dex group.

**Figure 6:**
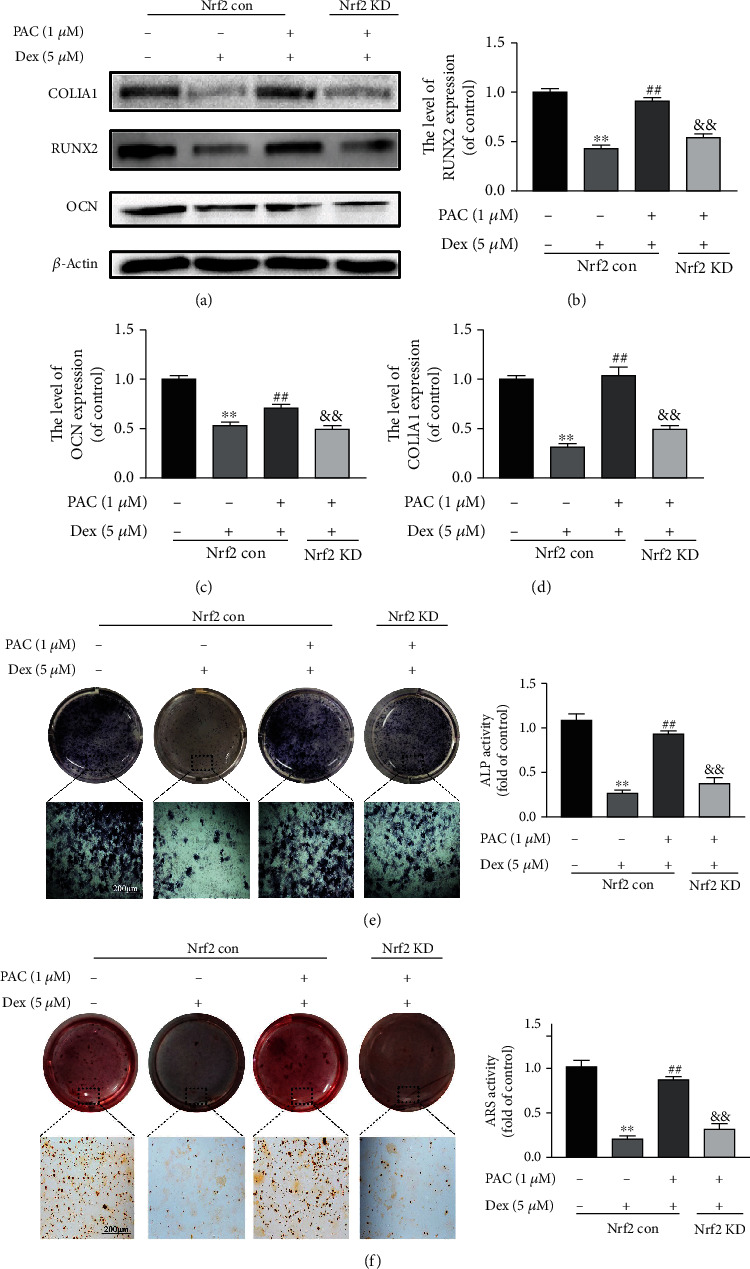
The Nrf2 pathway mediated the osteoprotective effects of PAC. Wild type (Nrf2 con) and Nrf2-knockdown osteoblasts were treated 1 *μ*M PAC with/out 5 *μ*M Dex pretreatment for 2 days. (a–d) Immunoblot showing RUNX2, COL1A1, and OCN in the differentially treated osteoblasts. Representative images showing ALP (e) staining on the 7^th^ day and (f) ARS staining on the 21^st^ day of osteogenic induction in differentially treated osteoblasts. Data are the averages ± SEM of 3 independent experiments. ^∗∗^*p* < 0.01 versus the untreated group, ^##^*p* < 0.01 versus the Dex group, ^&&^*p* < 0.01 versus the Dex+PAC group.

It was previously reported that PAC increased the nuclear translocation of Nrf2 in a lead-induced liver injury model, suggesting that the Nrf2 pathway likely mediates the protective effects of PAC [27]. As shown in Figures 5(a)–5(d), PAC treatment markedly reversed the inhibitory effect of Dex on the Nrf2 nuclear translocation and HO-1 expression and decreased the cytoplasmic Nrf2 levels in a dose-dependent manner (Figure 5(e)). Consistent with this, Nrf2 silencing abrogated the protective effects of PAC on the Dex-treated cells. Nrf2 knockdown partly decreased the expression levels of RUNX2, COL1A1, and OCN levels in the cells cotreated with PAC and Dex after 7 days of osteogenic induction (Figures 6(a)–6(d)) and reversed the beneficial effects of PAC on osteoblast differentiation and mineralization (Figures 6(e) and 6(f)). Taken together, PAC protect the osteoblast function by inducing the nuclear translocation of Nrf2 and activating its downstream pathway.

### 3.5. Pac Treatment Mitigated the Development of GIOP In Vivo

**Figure 7:**
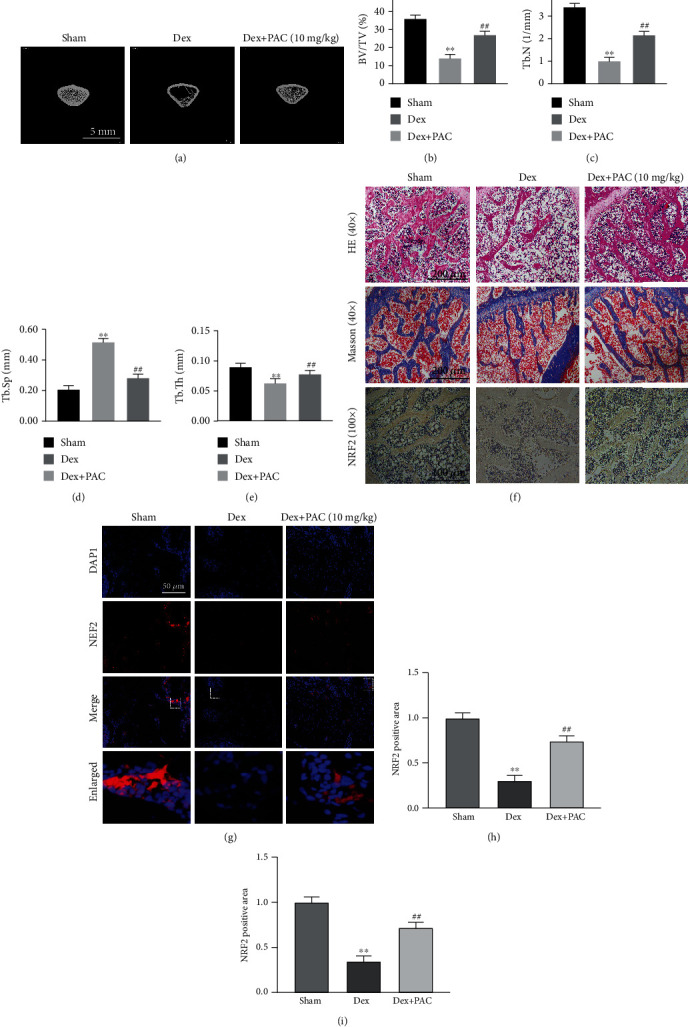
PAC treatment mitigated the development of GIOP in vivo. (a–e) Representative micro-CT images of the longitudinal and transverse sections of the distal femurs and the BV/TV, Tb.Sp, Tb.N, and Tb.Th values in the differentially treated animals. (f) Representative images of H&E staining, Masson's staining, and Nrf2 immunohistochemical (IHC) staining of the metaphyseal tissue of the thigh and (h) quantification of IHC. (g, i) Representative immunofluorescence image and quantification of the Nrf2 expression. Data are expressed as averages ± SEM of 3 times in duplicates. ^∗∗^*p* < 0.01 vs the SHAM group, ^##^*p* < 0.01 vs. the DEX group.

To further verify our assumption, the GIOP rat model was used in this study. The microstructure of the distal femur was determined by micro-CT scanning, and various parameters including BV/TV, Tb.Sp, Tb.Th, and Tb.N were calculated for each group (Figures 7(a)–7(e)). Compared to the untreated Dex-induced osteoporotic rats, 8 weeks of the PAC administration significantly increased BV/TV and Tb.N and decreased Tb.sp. Consistent with this, the histological examination of the distal femur sections indicated more numerous trabeculae in the PAC-treated versus untreated GIOP model. Moreover, the expression of Nrf2 in the distal femur was higher in the PAC-treated compared to the untreated animals (Figures 7(f)–7(i)). Taken together, PAC are potentially therapeutic against GIOP.

## 4. Discussion

**Figure 8:**
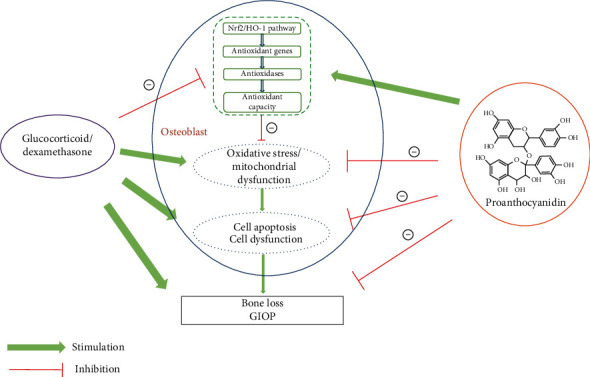
Mechanisms of the PAC action on GIOP. PAC reduces Dex-induced ROS accumulation and mitochondrial dysfunction in osteoblasts and prevents bone loss by activating the Nrf2 pathway.

In our study, we have proven for the first time that PAC can protect rat osteoblasts from oxidative stress, mitochondrial dysfunction, and osteogenic impairment induced by Dex. Mechanistically, as shown in Figure 8, PAC activated the antioxidant Nrf2 pathway and subsequently decreased ROS accumulation and mitochondrial superoxide levels in osteoblasts. In the in vivo GIOP model, PAC alleviated bone loss and upregulated the expression of Nrf2 in the distal femurs.

GCs are used to treat multiple inflammation-related diseases, and an estimated 1-2% of the population is currently receiving long-term GC treatment [28, 29]. Dex is a synthetic GC that accelerates osteoporosis and significantly increases the risk of fracture when used for an extended period of time [30]. Studies show that it can inhibit osteoblast proliferation and promote apoptosis [31, 32] by triggering oxidative stress and mitochondrial dysfunction. Li et al. found that Dex treatment increased intracellular ROS accumulation as well as the dissipation of MMP in MG63 cells [4]. The mitochondrial accumulation of ROS induced a vicious cycle of oxidative stress and mitochondrial dysfunction, which culminated in the activation of the apoptotic pathway [33]. Almeida et al. reported MMP loss and apoptosis in osteoblasts following Dex treatment [34]. Consistent with these studies, we found that Dex treatment led to osteoblast malfunction and apoptosis. Therefore, reducing the level of oxidative stress and mitochondrial dysfunction in osteoblasts is a potentially effective method for treating GIOP.

Proanthocyanidins (PAC) are a group of antioxidant polyphenolic flavonoids present in the dark pigmented fruits and vegetables [35]. Rodríguez-Pérez et al. reported that PAC prevent the H_2_O_2_-induced mitochondrial dysfunction and apoptosis in embryonic kidney cells [36]. In addition, the PAC administration protected against the iron overload-induced nephrotoxicity in rats [37]. Consistent with the above, we found that PAC significantly reduced Dex-induced oxidative stress and mitochondrial dysfunction in osteoblasts. Furthermore, PAC reduced the high rate of apoptosis in Dex-treated cells, downregulated the expression of apoptosis-associated protein Bax and cleaved caspase-3, and upregulated the anti-apoptosis protein Bcl-2. These results indicated that PAC may have an antiapoptosis effect to osteoblasts via attenuating the level of oxidative stress.

The major cause of bone loss in GIOP is the decrease in the osteoblast function. Dex inhibited osteoblast differentiation and accelerated the development of bone loss [4]. In addition, Dex disharmonizes organelle homeostasis within osteoblasts, thereby exacerbating its destiny of malfunction [32]. In the current study, we observed that Dex treatment reduced the expression of osteogenesis-related proteins RUNX2, COL1A1, and OCN, which was accompanied by the loss of osteogenic phenotype of ALP and ARS. Furthermore, coculture with PAC upregulated the expression of these proteins, which were suppressed by Dex and subsequently restored osteoblastogenesis in vitro. In vivo PAC treatment potently inhibited bone loss and microarchitechiture destruction induced by Dex. Therefore, PAC protect osteoblasts from Dex-induced oxidative stress and mitochondrial dysfunction and eventually restored osteoblastic function.

Nrf2 is considered an important transcription factor, and its activation can upregulate the expression of downstream genes of multiple antioxidant enzymes, such as HO-1, SOD, CAT, and GPx [5]. Consistent with previous reports that PAC activated the Nrf2 pathway, PAC facilitated the Nrf2 nuclear translocation and upregulated the expression of antioxidant enzymes in Dex-treated osteoblasts, which eventually lowered the level of oxidative stress and enhanced osteogenic function. In addition, knocking down Nrf2 abrogated the osteoprotective effects of PAC, which further indicated that the Nrf2 pathway mediated the PAC action. Consistent with the in vitro experiments, PAC prevented GIOP progression and restored the Nrf2 in situ expression in a rat model.

## 5. Conclusion

To sum up, our study proved that PAC protected osteoblasts against Dex-induced oxidative stress, mitochondrial dysfunction, and osteogenic impairment by activating the Nrf2 pathway. In addition, the PAC administration suppresses the process of the GIOP development and mitigates the GIOP development in vivo. However, a potential limitation of our study is that we did not compare the effect of PAC to several clinical medications used for the treatment in GIOP patients, whereas this comparison could help us evaluate PAC as a remedy for GIOP. Thus, this needs to be proved in future studies. Despite this limitation, our study has shown that Dex-induced oxidative stress and mitochondrial dysfunction are deleterious for the osteoblast differentiation and mineralization function. Meanwhile, these results, PAC supplementation may significantly improve clinical outcomes of GIOP patients.

## Data Availability

The data used to support the findings of this study are available from the corresponding author upon request.

## Supplementary Materials

Supplementary Materials Figure 1: effects of PAC on cell viability. Osteoblasts received different concentrations of PAC for 48 hours. The data in the figure represent the averages ± SEM of 3 times in duplicates. ^∗^*p* < 0.01, ^∗∗^*p* < 0.01 versus the untreated group. Figure 2: effects of different treating time of PAC on Dex-induced osteoblast viability. Percentage of viable cells received indicated treatment. The data in the figure represent the averages ± SEM of 3 times in duplicates. ^∗∗^*p* < 0.01 versus the untreated group, #*p* < 0.05, ##*p* < 0.01 versus the Dex group.
