# Supplementary material for: Proanthocyanidins-Mediated Nrf2 Activation Ameliorates Glucocorticoid-Induced Oxidative Stress and Mitochondrial Dysfunction in Osteoblasts
Source: Oxid Med Cell Longev. 2020 Sep 25;2020:9102012. doi: 10.1155/2020/9102012 (PMC7533007; doi:10.1155/2020/9102012)
Supplement: Supplementary Materials — Figure 1: effects of PAC on cell viability. Osteoblasts received different concentrations of PAC for 48 hours. The data in the figure represent the averages ± SEM of 3 times in duplicates. ∗p < 0.01, ∗∗p < 0.01 versus the untreated group. Figure 2: effects of different treating time of PAC on Dex-induced osteoblast viability. Percentage of viable cells received indicated treatment. The data in the figure represent the averages ± SEM of 3 times in duplicates. ∗∗p < 0.01 versus the untreated group, #p < 0.05, ##p < 0.01 versus the Dex group. [file 9102012.f1.pdf]

1. Materials and Methods

1.1 Cytotoxicity assay. The effect of PAC and Dex on osteoblast viability was determined using the Cell Counting Kit-8 (CCK-8) assay (MedChemExpress LLC; Monmouth Junction, NJ, USA). The cells were seeded in a 96-well plate at the density of 5×10^3 cells per well and received following treatment: ① PAC treatment (0, 0.25, 0.50, 1 ,5 and 10μM) for 48 hours; ② Osteoblasts were pretreated with 5μM Dexamethasone for 48 hours and then co-cultured with 1μM PAC for another indicated time (0, 12, 24, 48, 72, 96 hours), the medium was replaced with normal complete DMEM after aforementioned treating time. After treatment, 10μl CCK8 reagent was added to each well and the cells were incubated for another 2 hours. The absorbance or optical density (OD) at 450 nm was measured using Multiskan GO microdisk spectrophotometer (Thermo Fisher Science).

2. Results

2.1 1uM is the maximum safe concentration of PAC on osteoblast viability. After 48-hour treatment, we found 1uM PAC is the optimum concentration for osteoblast viability.

2.2 Prolonged treating time did not leads to a higher osteoblast viability. As we did in our previous experiment, we found there was no significant difference in cell viability between 48 hours and a prolonged treating time. To this end, we selected 48 hours as our PAC treating time.

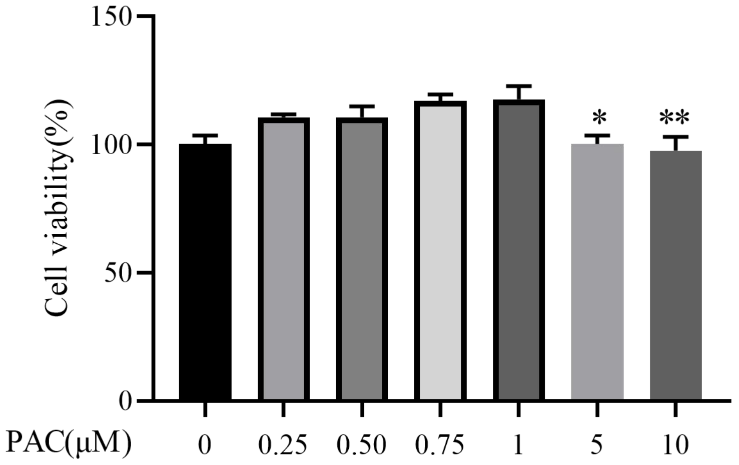

Figure 1: Effects of PAC on cell viability. Osteoblasts received different concentrations of PAC for 48 hours. The data in the figure represent the averages ± SEM of 3 times in duplicates. *p < 0.01, **p < 0.01 versus untreated group.

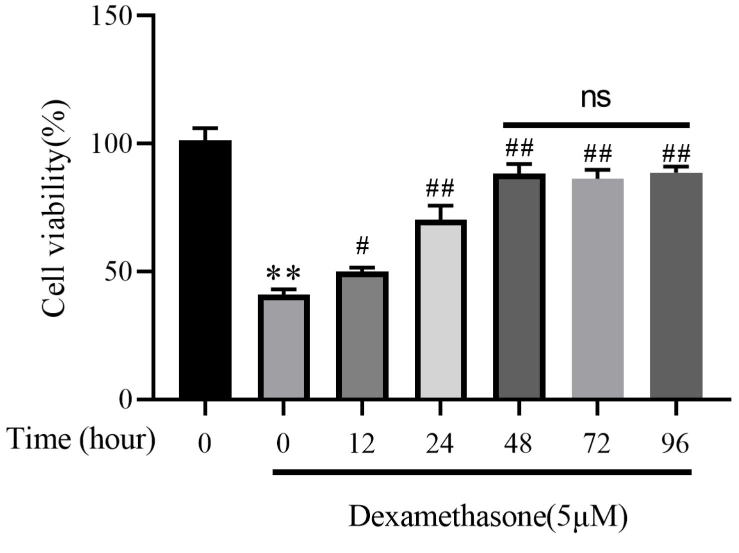

Figure 2: Effects of different treating time of PAC on Dex-induced osteoblast viability. Percentage of viable cells received indicated treatment. The data in the figure represent the averages ± SEM of 3 times in duplicates. **p < 0.01 versus untreated group, $^{\#}p < 0.05$, $^{\#\#}p < 0.01$ versus Dex group.
